# Modest Gains After an 8-Week Exercise Program Correlate With Reductions in Non-traditional Markers of Cardiovascular Risk

**DOI:** 10.3389/fcvm.2021.669110

**Published:** 2021-06-17

**Authors:** Grace Liang, Xianxi Huang, James Hirsch, Sanjeev Mehmi, Holly Fonda, Khin Chan, Ngan F. Huang, Oliver Aalami, Victor F. Froelicher, David P. Lee, Jonathan Myers, Andrew S. Lee, Patricia K. Nguyen

**Affiliations:** ^1^Division of Cardiovascular Medicine, Stanford University, Stanford, CA, United States; ^2^Department of Critical Care Medicine, The First Affiliated Hospital of Shantou University Medical College, Shantou, China; ^3^Stanford Cardiovascular Institute, Stanford, CA, United States; ^4^Cardiology Section, Department of Veteran Affairs, Palo Alto, CA, United States; ^5^Department of Cardiovascular Surgery, Stanford University, Stanford, CA, United States; ^6^Vascular Surgery Section, Department of Veteran Affairs, Palo Alto, CA, United States; ^7^Department of Pathology, Stanford University, Stanford, CA, United States

**Keywords:** exercise, coronary artery disease, biomarkers, thrombosis, inflammation

## Abstract

**Background:** Although engaging in physical exercise has been shown to reduce the incidence of cardiovascular events, the molecular mechanisms by which exercise mediates these benefits remain unclear. Based on epidemiological evidence, reductions in traditional risk factors only accounts for 50% of the protective effects of exercise, leaving the remaining mechanisms unexplained. The objective of this study was to determine whether engaging in a regular exercise program in a real world clinical setting mediates cardiovascular protection via modulation of non-traditional risk factors, such as those involved in coagulation, inflammation and metabolic regulation.

**Methods and Results:** We performed a prospective, cohort study in 52 sedentary patients with cardiovascular disease or cardiovascular risk factors at two tertiary medical centers between January 1, 2016 and December 31, 2019. Prior to and at the completion of an 8-week exercise program, we collected information on traditional cardiovascular risk factors, exercise capacity, and physical activity and performed plasma analysis to measure levels of fibrinolytic, inflammatory and metabolic biomarkers to assess changes in non-traditional cardiovascular risk factors. The median weight change, improvement in physical fitness, and change in physical activity for the entire cohort were: −4.6 pounds (IQR: +2 pounds, −11.8 pounds), 0.37 METs (IQR: −0.076 METs, 1.06 METs), and 252.7 kcals/week (IQR: −119, 921.2 kcals/week). In addition to improvement in blood pressure and cholesterol, patients who lost at least 5 pounds, expended at least 1,000 additional kcals/week, and/or achieved ≥0.5 MET increase in fitness had a significant reduction in plasminogen activator inhibitor-1 [9.07 ng/mL (95% CI: 2.78–15.35 ng/mL); *P* = 0.026], platelet derived growth factor beta [376.077 pg/mL (95% CI: 44.69–707.46 pg/mL); *P* = 0.026); and angiopoietin-1 [(1104.11 pg/mL (95% CI: 2.92–2205.30 pg/mL); *P* = 0.049)].

**Conclusion:** Modest improvements in physical fitness, physical activity, and/or weight loss through a short-term exercise program was associated with decreased plasma levels of plasminogen activator inhibitor, platelet derived growth factor beta, and angiopoietin, which have been associated with impaired fibrinolysis and inflammation.

## Translational Perspective

This is a prospective study that compares the effects of exercise on non-traditional and traditional cardiovascular risk factors. In 52 patients, we found that weight loss of ≥5 pounds, an improvement in physical fitness of ≥0.5 METs, and an increase in physical activity of ≥1,000 kcals/week, achieved through a short-term exercise program resulted in decreases in plasma levels of plasminogen activator inhibitor-1, platelet derived growth factor beta-1, and angiopoetin-1, which have been associated fibrinolysis and inflammation. These findings suggest that modest improvements in weight loss, physical fitness, and physical activity levels may reduce cardiovascular risk through modulation of factors involved in coagulation and inflammation.

## Introduction

It is well-known that exercise reduces morbidity and mortality associated with cardiovascular disease. Exercise and physical activity are, thus, Class I indications for the prevention of atherosclerotic cardiovascular disease (ASCVD) (1–3). Performing physical activity assessment and dietary counseling during clinical visits is considered an important strategy to encourage patients to exercise and lead a healthy lifestyle. Although there is a defined minimum amount of recommended activity (e.g., 150 min of moderate intensity physical activity or 75 min of vigorous activity) to reduce the risk of ASCVD (1–3), patients who engage in lower levels of physical activity than the minimum recommended amount have also been found to have significantly lower risk of coronary heart disease (4), suggesting that some physical activity is better than none.

The mechanisms by which exercise mediates cardio-protection remain poorly understood. While physical fitness and activity can reduce cardiovascular risk by as much as 30–50%, changes in individual traditional risk factors are small, with reported decreases of 5% for blood lipids (5), 3–5 mmHg for blood pressure (6), and 1% for hemoglobin A1C (7). Although previous studies have demonstrated exercise reduces novel risk factors in addition to traditional risk factors, these studies have been limited to the measurement of only a handful of biomarkers (8–12) and a reliance on self-reported measures of physical activity, fitness or weight loss (8–10, 13).

To address this knowledge gap, we measured levels of novel and traditional biomarkers associated with cardiovascular disease risk in a cohort of 52 patients recruited from a real world clinical setting. Our objective was to determine whether exercise-induced improvement in weight loss, physical fitness, and activity alone or in combination, correlated with changes in traditional and non-traditional biomarkers of cardiovascular risk.

## Methods

### Study Design

We conducted a prospective, cohort study that consecutively enrolled patients who were advised by their cardiologist to engage in healthy lifestyle behaviors for primary or secondary prevention of cardiovascular disease. The objective of the study was to correlate improvement in weight loss, physical fitness, and physical activity achieved through a short-term exercise program with traditional and non-traditional biomarkers. We hypothesized that exercise modulates these non-traditional markers of risk independent of its effects on traditional risk factors. The study was approved by the Institutional Human Research Ethics Committee and confirmed to the Declaration of Helsinki. All participants provided written, informed consent before inclusion into the study. Anonymized data supporting the findings in this article are available from the corresponding author upon reasonable request.

### Patient Population

**Figure 1:**
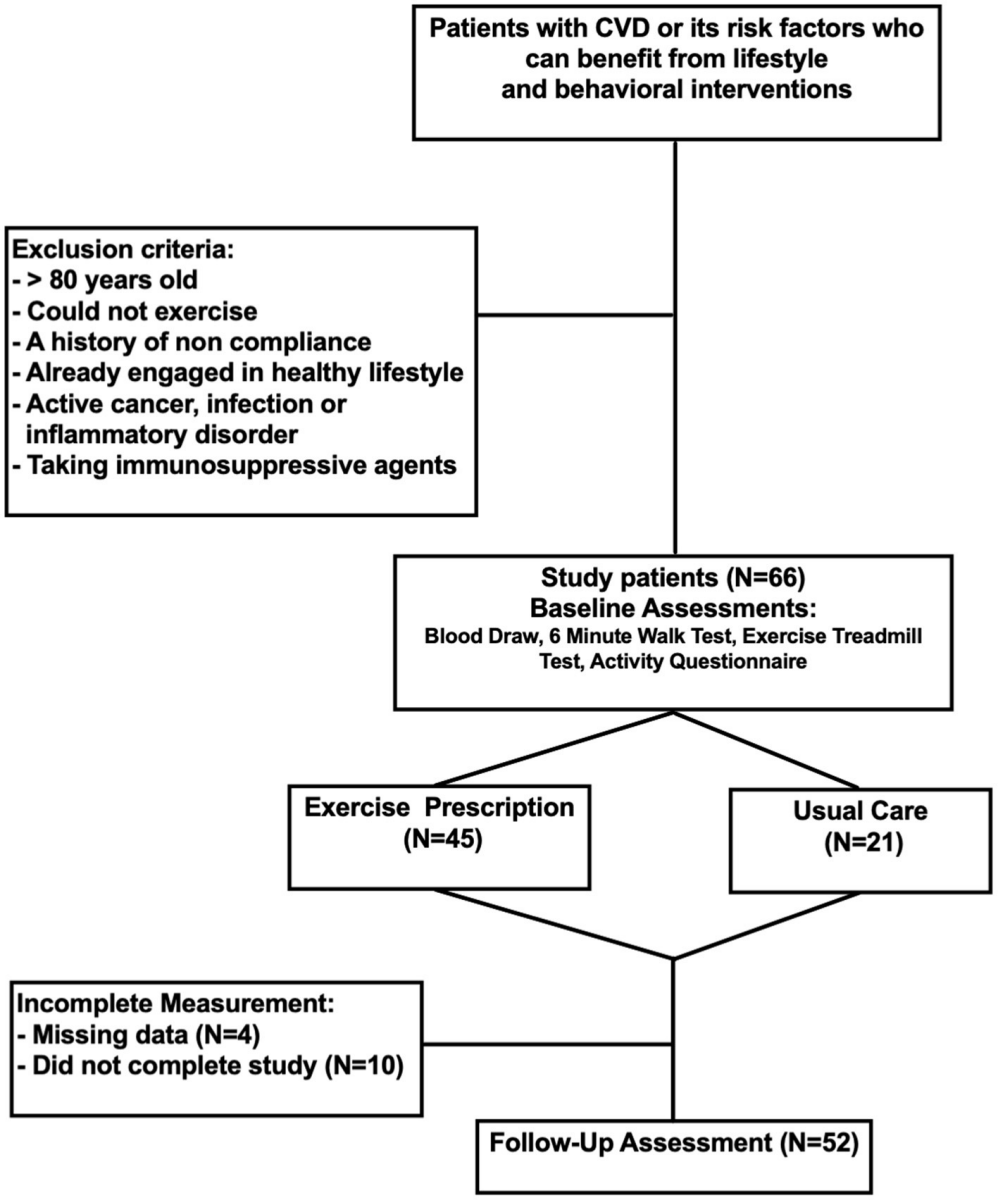
Schematic of study design.

Between January 1, 2016 and December 31, 2019, we recruited 66 patients who were advised by their cardiologist to engage in healthy lifestyle modifications for primary and secondary prevention of cardiovascular disease at Stanford Hospital and the Veterans Affairs Palo Alto Health Care System. Patients were excluded if they were already adhering to the recommended weekly activity levels as defined by the American Heart Association, could not exercise, had a history of non-compliance, a history of active cancer or inflammatory disorder, or were taking immunosuppressive agents. Patients that had any changes in medications during the study period that could affect blood pressure, lipids, inflammation, and coagulation were excluded from the study. Forty-five patients met the inclusion and exclusion criteria and completed a supervised exercise program (e.g., formal in-person cardiac rehabilitation program, or remotely monitored program with active weekly feedback) and 21 patients participated in an unsupervised program (e.g., exercised on their own with advice from study staff. Assessments were performed at baseline and after subjects completed 8 weeks of their program. Of the 66 patients initially recruited, 52 completed both baseline and terminal assessments and were included in the analysis (Figure 1).

### Traditional Cardiovascular Risk Assessment

Patient demographics, medical history (e.g., hypertension, diabetes, dyslipidemia, family history of premature coronary artery disease, tobacco use), and medication use were collected through chart review. Fasting lipid profile, fasting glucose, glycosylated hemoglobin, lipoprotein A, apolipoprotein B, and high sensitivity C-reactive protein levels were also measured.

### Assessment of Physical Fitness and Physical Activity Levels

Exercise capacity was determined from cardiopulmonary exercise testing using standard protocols. Briefly, subjects were tested using an individualized ramp treadmill protocol with initial ramp rate determined by a questionnaire to estimate exercise capacity (14, 15). The treadmill test was symptom-limited; in the absence of angina, subjects were encouraged to reach maximal effort (defined as exhaustion, a score ≥18 on the Borg scale, or both); target heart rates were not used as testing endpoints. Treadmill tests were performed with breath by breath gas exchange measurements using a CosMed Quark system (Rome, Italy) to determine peak VO_2_, which was then converted to measured metabolic equivalents (METs). Physical fitness was also assessed by six-minute walk tests, which were performed in accordance with guidelines set forth by the American Thoracic Society (16). To assess physical activity levels, subjects completed the Veterans Physical Activity Questionnaire (17), a well-validated tool to assess weekly physical activity measured in MET-hours that was then converted to weekly kilocalories using an algorithm proposed by the American College of Sports Medicine (ACSM) (18).

### Assessment of Quality of Life

The MacNew Heart Disease Health-Related Quality of Life Questionnaire was administered on all patients before and after engaging in the 8-week healthy lifestyle program. The MacNew is designed to assess a patient's feelings about how cardiovascular disease affects their daily function and produces a global score as well sub-scores to evaluate physical limitation, emotional function and social function (19).

### Biomarker Assessments

Blood samples for biomarker analysis were processed within 1–4 h of collection as follows: samples were collected in BD Vacutainer® CPT™ Mononuclear Cell Preparation Tubes with sodium citrate. Samples were then spun down in a Beckman Coulter Allegra [X-12R] centrifuge at 1800RCF for 20 min with the acceleration set at 9 and deceleration set to 0. Plasma was collected from the CPT tubes after separation from the PBMC layer and aliquoted into cryo-vials for storage at −80°C.

Based on previous studies, we defined subjects who lost ≥5 pounds (20), whose METs increased by 0.5 or greater (21), or had physical activity increase by 1,000 kcals or greater per week (17) as having successfully completed the 8-week program. Otherwise, patients were deemed unsuccessful. The most successful patients were those that were at the top tertile in weight loss, MET increase or physical activity. The least successful were those who were at the top tertile of weight gain, MET decrease or reduction in physical activity. In patients who were deemed the most successful (*n* = 15) and the least successful (*n* = 6), plasma was used to measure protein levels for 184 biomarkers including those associated with coagulation, inflammation and metabolism via the OLINK Proseek Multiplex Assay for Cardiovascular II and Cardiovascular III Panels (Olink Proteomics AB, Uppsala, Sweden). Protein names and abbreviations are listed in Supplementary Table 1. Protein levels for the 184 biomarkers were quantified by real-time PCR using a proximity extension assay utilizing oligonucleotide-labeled antibody probe pairs. Quality control and data validation were performed using OLINK's standard quality control pipeline. All Olink samples were analyzed in a blinded fashion.

Findings from this comprehensive screen were confirmed by performing the ELISA assay using the following kits based on manufacturer's protocol: Human PAI-1 Standard ABTS ELISA Development Kit (PeproTech, Rocky Hill, New Jersey), Human PDGF-Beta-1 Standard ABTS ELISA Development Kit (Peprotech, Rocky Hill, New Jersey), and Human Angiopoietin-1 ELISA Kit (ThermoScientific, Frederick, Maryland). Analysis of selected proteins by ELISA was performed on the entire cohort. All ELISA samples were performed in triplicate and analyzed in a blinded fashion.

### Statistical Analysis

Data are presented as median with interquartile range for continuous variables or numbers (n) and percentage (%) for categorical variables. Results from the six-minute walk test and cardiopulmonary exercise tests were converted to METs (22). In subjects unable to complete a valid treadmill test, METs from the six-minute walk test were used in the analysis (*n* = 5). As shown in previous studies (23), a significant correlation was found between METs obtained from the six-minute walk test and cardiopulmonary exercise test for patients who completed both assessments (Spearman's *r* = 0.32, *p*-value = 0.039, Supplementary Figure 1).

Unpaired and paired Students' *t*-tests, Mann Whitney *U* tests, and Chi-Square tests were used as appropriate. Results from Olink assays were reported in NPX (normalized protein expression), which is expressed in log2 scale and subsequently converted to fold change for comparison against ELISA results (https://www.olink.com/content/uploads/2019/02/Technical-Summary-Olink.pdf). A multivariate logistic regression analysis was performed using demographics, clinical parameters, and improvement in biomarkers as independent predictors of a successful or unsuccessful outcome. All *p*-values were adjusted for multiple comparisons using the Bonferroni correction method.

A propensity score model was used to balance the effect of covariates between the successful and unsuccessful groups. Patients who were defined as successful and unsuccessful were placed in the “treatment” or “control” group, respectively. An estimation of treatment effects was calculated using propensity-score matching as the estimator, matching as the outcome model, and logistic regression as the treatment model. The following outcomes were entered into the model: (1) change in plasminogen activator inhibitor-1 (PAI-1) between the first and second timepoint; (2) change in platelet derived growth factor beta-1 (PDGF-beta-1) between the first and second timepoint; and (3) change in angiopoetin-1 (ANG-1) between the first and second timepoint. Only values measured by ELISA were entered into the model. Covariates entered into the model included age (continuous), sex, race (categorical), smoking status (never, former, or current), and cardiovascular risk factors (e.g., systolic blood pressure, diastolic blood pressure, lipid profile, lipoproteins, hemoglobin A1C, and C-reactive protein). A *p*-value < 0.05 was considered significant with adjustment for multiple comparisons if appropriate.

## Results

### Patient Characteristics

Baseline subject characteristics are described in Table 1. Median age was 67.0 years (IQR 58.3, 70.8 years), 92% were male, 63% were white, and the median BMI was 33.5 kg/m^2^ (IQR 28.3, 38.6 kg/m^2^). Ninety-two percent of participants were obese, 42% were diabetic, 69% had hypertension, 65% had hypercholesteremia, and 73% had a history of coronary artery disease. A majority of subjects were on statins (75%), aspirin (69%), and beta blockers (56%) at baseline. There were no significant differences in patient characteristics between those who successfully completed their exercise programs and those who did not.

**Table 1:** Demographic and clinical factors.

	**All completed patients**	**Successful patients**	**Unsuccessful patients**	***P*-value**
**Demographics**	***n* = 52**	***n* = 35**	***n* = 17**	
Age	67.0 (58.3, 70.8)	67.0 (59.0, 69.0)	69.0 (57.5, 74.5)	0.094
Male	0.92 (48)	0.91 (32)	0.94 (16)	0.438
Ethnicity				
White	0.63 (33)	0.60 (21)	0.71 (12)	0.269
Hispanic/Latino)	0.10 (5)	0.11 (4)	0.06 (1)	0.374
African American	0.08 (4)	0.09 (3)	0.06 (1)	0.438
Asian	0.10 (5)	0.06 (2)	0.18 (3)	0.244
Native American	0.02 (1)	0.03 (1)	0.00 (0)	0.434
Pacific Islander	0.08 (4)	0.11 (4)	0.00 (0)	0.254
Clinical characteristics				
BMI	33.5 (28.3, 38.6)	34.0 (28.2, 43.0)	32.0 (28.5, 37.1)	0.209
Weight	219.5 (187.8, 262.8)	216.0 (194.0, 266.5)	220.0 (176.0, 262.0)	0.235
Medical history				
Obesity	0.92 (48)	0.94 (33)	0.88 (15)	0.363
Diabetes	0.42 (22)	0.40 (14)	0.47 (8)	0.341
Hypertension	0.69 (36)	0.71 (25)	0.65 (11)	0.348
Heart failure	0.10 (5)	0.06 (2)	0.18 (3)	0.244
Hypercholesteremia	0.65 (34)	0.63 (22)	0.71 (12)	0.327
Smoking	0.17 (9)	0.14 (5)	0.24 (4)	0.296
COPD	0.04 (2)	0.06 (2)	0.00 (0)	0.369
CKD	0.19 (10)	0.14 (5)	0.29 (5)	0.158
CAD	0.73 (38)	0.71 (25)	0.76 (13)	0.385
Angina	0.10 (5)	0.09 (3)	0.12 (2)	0.426
Myocardial Infarction	0.21 (11)	0.26 (9)	0.12 (2)	0.209
Past PCI	0.37 (19)	0.37 (13)	0.35 (6)	0.457
Recent PCI	0.23 (12)	0.23 (8)	0.24 (4)	0.484
Past valve repair	0.10 (5)	0.06 (2)	0.18 (3)	0.244
Recent valve repair	0.06 (3)	0.03 (1)	0.12 (2)	0.303
Past CABG	0.15 (8)	0.11 (4)	0.24 (4)	0.241
Recent CABG	0.00 (0)	0.00 (0)	0.00 (0)	0.5
Baseline medications				
Statin	0.75 (39)	0.77 (27)	0.71 (12)	0.305
Aspirin	0.69 (36)	0.71 (25)	0.65 (11)	0.348
P2Y12 inhibitor	0.38 (20)	0.46 (16)	0.24 (4)	0.099
Warfarin	0.12 (6)	0.11 (4)	0.12 (2)	0.492
DOACs	0.04 (2)	0.00 (0)	0.12 (2)	0.247
ACE inhibitors	0.37 (19)	0.40 (14)	0.29 (5)	0.269
ARBs	0.17 (9)	0.17 (6)	0.18 (3)	0.488
Beta blockers	0.56 (29)	0.57 (20)	0.53 (9)	0.404
CCB	0.13 (7)	0.11 (4)	0.18 (3)	0.359
Diuretics	0.33 (17)	0.34 (12)	0.29 (5)	0.389
Hydralazine	0.06 (3)	0.06 (2)	0.06 (1)	0.496
Insulin	0.10 (5)	0.09 (3)	0.12 (2)	0.426
Metformin	0.27 (14)	0.26 (9)	0.29 (5)	0.415
Oral anti-glycemics	0.06 (3)	0.06 (2)	0.06 (1)	0.496
Nitrates	0.17 (9)	0.23 (8)	0.06 (1)	0.162
Nitroglycerin	0.23 (12)	0.23 (8)	0.24 (4)	0.484

### Changes in Weight, Physical Fitness, and Physical Activity

**Figure 2:**
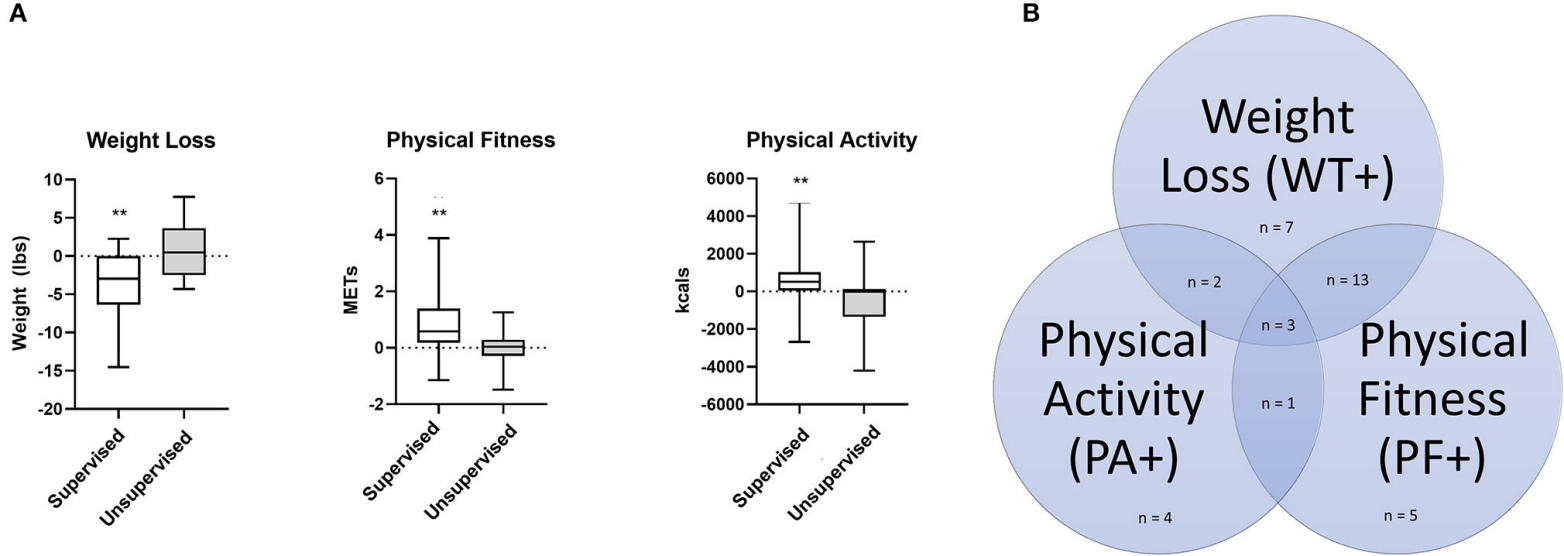
**(A)** Bar graphs showing in weight loss, physical fitness, and physical activity in patients engaged in supervised (*n* = 36) and unsupervised (*n* = 16) programs. **P-value ≤ 0.01. For supervised group: weight loss, *p* = 0.0029; METs change, *p* = 0.0025; kcal change, *p* = 0.0111; Mann-Whitney *U* Test. **(B)** Venn diagram showing the number of patients who met the pre-defined criteria for success: Weight loss ≥5 pounds, increase in physical activity of greater or equal to 1,000 kcals per week, and/or improvement in metabolic equivalents of greater or equal to 0.5 METs. Patients who met at least one criteria were deemed successful.

Of 66 enrolled patients, 52 completed baseline and terminal testing. The median weight change, improvement in physical fitness, and change in physical activity for the entire cohort were −4.6 pounds (IQR: +2 pounds, −11.8 pounds), 0.37 METs (IQR: −0.076 METs, 1.06 METs), and 252.7 kcals/week (IQR: −119, 921.2 kcals/week), respectively. Patients in the supervised exercise program showed significant improvement in weight (−2.96 vs. 0.46 pounds, *p* = 0.003), physical fitness (0.59 vs. 0.04 METs, *p* = 0.002), and physical activity (510.0 vs. −13.1 kcals/week, *p* = 0.01) compared to the unsupervised group (Figure 2A). Sixty-seven percent of patients (35/52) successfully achieved one of the pre-defined cut-offs in weight, physical fitness or physical activity that have been previously defined as thresholds to improve cardiovascular health after engaging in short-term exercise training, and only three patients met all three criteria (Figure 2B, Table 2) (17, 20, 21). Not surprisingly, of patients who were successful, the majority (77%, 27/35) were enrolled in the supervised program. Of the total supervised patients who completed the study (*n* = 36), remotely supervised patients were more successful than subjects who completed a program in-person [92% (22/24) vs. 42% (5/12), *p* = 0.001].

**Table 2:** Changes in weight loss, physical fitness, and physical activity pre- and post-intervention.

	**Successful patients**			**Unsuccessful patients**		
	**Pre**	**Post**	***P*-value**	**Pre**	**Post**	***P*-value**
Weight (lbs)	217.1 (194, 260.3)	206.5 (184.5, 262.5)	<0.0001	219.5 (176, 256)	219 (182, 256)	0.049
BMI	34.0 (29.1, 41.6)	32.9 (27.8, 41.0)	<0.0001	31.5 (28.9, 37.0)	32.9 (28.9, 37.3)	0.043
6MW (ft)	1362.5 (1200, 1525)	1445 (1312.5, 1694.8)	0.006	1300 (1075, 1380)	1250 (800, 1600)	0.14
METS	5.7 (4.3, 6.7)	6.23 (4.71, 8.24)	0.004	5.61 (3.87, 7.20)	5.34 (3.66, 7.34)	0.76
VO_2_ max (L/min)	20.1 (17.7, 23.6)	23.4 (19.9, 29.8)	0.001	19.8 (14.3, 25.7)	20.6 (15.9, 25.7)	0.89
ETT Time (sec)	486 (380, 631)	570 (486, 740)	0.002	450 (410, 540)	420 (375, 552)	0.42
Physical activity (kcals/week)	1427.3 (627.2, 2408)	1611.4 (977.2, 2867.9)	0.04	1476.3 (576.8, 2333.8)	1381.8 (698.6, 1966.3)	0.31

As shown in Table 2, compared to baseline, successful patients had significant improvements in weight (217.1 vs. 206.5 pounds, *p* < 0.0001) and physical fitness as measured by the six-minute walk test (1,362.5 vs. 1,445 feet, *p* = 0.006), METs (5.7 vs. 6.23 METs, *p* = 0.004), VO_2_ max (20.1 vs. 23.4 liters/min, *p* = 0.001), treadmill time (486 vs. 570 s, *p* = 0.002), and weekly physical activity (1,427.3 vs. 1,611.4 kcals/week, *p* = 0.04). In contrast, unsuccessful patients had no significant improvement in weight loss, physical fitness, or physical activity. The median change in weight loss [−7.6 pounds (IQR: −8.2, 21.2 pounds) vs. +7.3 pounds (IQR: +8.0 pounds, −0.55 pounds), *p* < 0.0001] and physical fitness [0.95 METs [IQR: 0.44 METs, 2.3 METs) vs. 0.04 METs (IQR: 0.19 METs, 0.86 METs), *p* = 0.003] was significantly different between the successful and unsuccessful groups. Although the median change in physical activity was not significantly different between the two groups [273.5 kcal/week (−101.3, 1090.2 kcal/week) vs. 112.7 kcal/week (−204.8, 721.3 kcal/week)], 27.8% (10/35) of patients in the successful group increased their physical activity level by at least 1,000 kcals/week.

### Changes in Blood Pressure, Lipids, Metabolic Parameters, and High-Sensitivity C-Reactive Protein Post-Intervention

Successful patients had a significant improvement in diastolic blood pressure (80 vs. 75 mmHg, *p* = 0.0025) and a non-significant decrease in systolic blood pressure (134 vs. 126 mmHg, *p* = 0.17). Total cholesterol (146 vs. 131 mg/dL, *p* = 0.03) and LDL (85 vs. 67.5 mg/dl, *p* = 0.02) were also significantly lower. Although anthropometric measures including waist and hip circumference (44.8 vs. 41.5 inches, *p* = 0.005; and 42.0 vs. 41.0, inches *p* = 0.008) as well as fasting glucose (105.5 vs. 97.5 md/dl, *p* = 0.03) were significantly improved, hemoglobin A1C (6.0 vs. 5.7%, *p* = 0.38) was not significantly different. Successful patients had a non-significant decrease in C-reactive protein post-intervention (1.9 vs. 1.05 mg/dL, *p* = 0.69). Unsuccessful patients, however, exhibited a non-significant increase in LDL and C-reactive protein (Table 3).

**Table 3:** Changes in blood pressure, lipids, metabolic parameters, and high-sensitivity C-reactive protein.

	**Successful patients**			**Unsuccessful patients**		
	**Pre**	**Post**	***P*-value**	**Pre**	**Post**	***P*-value**
Systolic blood pressure (supine)	134 (117, 136)	126 (122, 136)	0.165	128 (122, 132)	120 (118, 130)	0.07
Diastolic blood pressure (supine)	80 (76, 88)	75 (70, 80)	0.0025	78 (70,82)	77 (68.5, 79)	0.5782
Total cholesterol (mg/dL)	146 (120, 172)	131 (125, 147)	0.03	150 (123, 162)	150 (125, 186)	0.22
Triglyceride (mg/dL)	99 (76, 127)	87 (72, 111)	0.49	125 (99, 172)	105 (70, 142)	0.09
HDL (mg/dL)	46 (35, 53)	45 (35, 55)	0.97	44.5 (35.5, 56)	45.5 (37, 54.5)	0.23
LDL (mg/dL)	85 (60, 109)	67.5 (52, 92)	0.015	77 (67, 89)	84 (74, 96)	0.29
Total cholesterol/HDL ratio	3.41 (2.62, 3.84)	2.95 (2.41, 3.66)	0.38	3.03 (2.59, 3.49)	3.41 (2.62, 3.84)	0.28
Apolipoprotein B (mg/dL)	74 (60, 89)	70 (53, 80)	0.098	71 (64, 86)	71 (65, 80)	0.68
Lipo A (nmol/L)	43 (14, 145)	44.8 (20, 151)	0.19	19 (10, 110)	15 (10, 38)	0.8
Waist (in.)	44.8 (40.5, 50)	41.5 (39.5, 48.5)	0.005	41 (37, 47.5)	40 (36.8, 46.8)	0.75
Hip (in.)	42 (40.0, 51.0)	41.0 (39.5, 48)	0.008	41.3 (36.5, 47.5)	40.5 (36.5, 45.5)	0.45
W/H	1.03 (0.98, 1.07)	1.02 (0.99, 1.05)	0.31	1.01 (0.95, 1.05)	1.02 (0.94, 1.04)	0.41
Hga1c (mg/dL)	6 (5.7, 6.5)	5.7 (5.6, 6.5)	0.38	5.95 (5.45, 7.35)	5.8 (5.6, 7.4)	0.59
Fasting glucose (mg/dL)	105.5 (96, 116)	97.5 (93, 105)	0.03	101 (93, 137)	96 (87, 118)	0.75
CRP (mg/dL)	1.9 (0.9, 2.4)	1.05 (0.55, 3.25)	0.69	0.6 (0.4, 3.6)	1 (0.5, 5.5)	0.23

### Changes in MacNew Quality of Life Scores

Although there was a trend toward improvement in emotional, physical, social, and global MacNew scores, there was no significant changes in quality of life scores in both the successful and unsuccessful groups (Table 4).

**Table 4:** Changes in MacNew quality of life measures.

	**Successful patients**			**Unsuccessful patients**		
	**Pre**	**Post**	***P*-value**	**Pre**	**Post**	***P*-value**
Emotional	5.96 (5.17, 6.39)	5.64 (4.36, 6.5)	0.9	5.60 (4.79, 6.21)	6.11 (5.61, 6.54)	0.76
Physical	5.36 (4.9, 5.86)	5.96 (4.79. 6.36)	0.058	5.64 (4.82, 6.07)	6.14 (5.46, 6.57)	0.127
Social	5.96 (4.81, 6.61)	6.30 (5.38, 6.77)	0.051	5.96 (5.38, 6.31)	6.73 (5.65, 6.88)	0.22
Global	5.89 (4.96, 6.22)	5.93 (4.63, 6.33)	0.083	5.39 (5.04, 0.148)	6.22 (5.67, 6.59)	0.23

### Changes in Novel Biomarkers of Cardiovascular Risk

**Figure 3:**
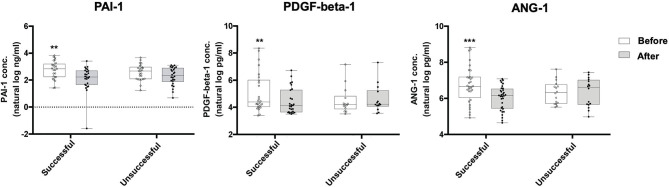
Box and whisker plots comparing ELISA results for the levels of PAI-1, PDGF-beta-1, and ANG-1 in successful and unsuccessful patients before and after engaging in an 8-week exercise program. Successful patients (*n* = 35) had a significant decrease in PAI-1 (16.64 vs. 9.38 ng/mL, *p* = 0.0002), PDGF-beta-1 (76.71 vs. 60.32 pg/mL, *p* = 0.0043), and ANG-1 (788.89 vs. 475.28 pg/mL, *p* = 0.0001), whereas unsuccessful patients (*n* = 17) either showed a non-significant decrease (PAI-1, 14.47 vs. 10.33 ng/mL, *p* = 0.098) or had an increase (PDGF-beta-1, 67.77 vs. 70.82 pg/mL, *p* = 0.42; ANG-1, 539.4 vs. 693.55 pg/mL, *p* = 0.776) in these markers. Values on graph shown in natural log. Wilcoxon signed-rank test; **P-value ≤ 0.01; ***P ≤ 0.001. Box defines the first and third quartiles; whiskers show minimum and maximum.

Of the 184 biomarkers of cardiovascular risk that we measured, the most significant difference between the 15 most successful patients and the 6 least successful patients was found in PAI-1 (fold change compared to baseline of −2.02 in successful group, *p* = 0.004), PDGF-beta-1 (fold change compared to baseline of 2.03 in the unsuccessful group, *p* = 0.027), and ANG-1 (fold change compared to baseline of 2.07 in the unsuccessful group, *p* = 0.019) (Table 5). To confirm these findings, ELISA was performed in the entire cohort; successful patients had a significant decrease in PAI-1 (16.64 vs. 9.38 ng/mL, *p* = 0.0002), PDGF-beta-1 (76.71 vs. 60.32 pg/mL, *p* = 0.0043), and ANG-1 (788.89 vs. 475.28 pg/mL, *p* = 0.0001), whereas unsuccessful patients either showed a non-significant decrease (PAI-1, 14.47 vs. 10.33 ng/mL, *p* = 0.098) or had an increase (PDGF-beta-1, 67.77 vs. 70.82 pg/mL, *p* = 0.42; ANG-1, 534.9 vs. 693.55 pg/mL, *p* = 0.776) (Figure 3, Table 6). Importantly, there was significant correlation between biomarkers measured by the Olink and ELISA Assays (Supplementary Figure 2). On multivariate logistic regression analysis, changes in PAI-1, PDGF-beta-1, and ANG-1 were not independent from improvement in blood pressure, lipid profile, and glucose control, suggesting that improvement in these biomarkers may be a secondary effect.

**Table 5:** Biomarker levels measured by OLINK pre- and post-intervention.

	**Successful patients**				**Unsuccessful patients**			
	**Pre**	**Post**	**Fold change**	***P*-value**	**Pre**	**Post**	**Fold change**	***P*-value**
PAI-1	5.17 (4.72, 5.66)	3.92 (3.45, 4.72)	−2.02	0.004	4.23 (3.89, 4.80)	4.93 (3.59, 5.43)	0.32	0.212
PDGF-beta-1	8.20 (7.21, 10.74)	7.51 (6.81, 8.22)	−1.16	0.127	7.49 (6.66, 7.64)	8.68 (7.49, 9.36)	2.03	0.027
ANG-1	7.42 (6.64, 9.46)	6.74 (6.51, 7.42)	−1.15	0.089	6.88 (6.28, 7.36)	8.00 (7.18, 8.67)	2.07	0.019

**Table 6:** Biomarker levels measured by ELISA pre- and post-intervention.

	**Successful patients**			**Unsuccessful patients**		
	**Pre**	**Post**	***P*-value**	**Pre**	**Post**	***P*-value**
PAI-1 (ng/mL)	16.64 (9.20, 22.98)	9.38 (5.32, 14.55)	0.0002	14.47 (8.02, 20.0)	10.33 (7.22, 19.3)	0.098
PDGF-beta-1 (pg/mL)	76.71 (55.41, 386.59)	60.32 (38.73, 194.28)	0.0043	67.77 (58.16, 109.45)	70.82 (63.49, 174.18)	0.42
ANG-1 (pg/mL)	788.89 (595.27, 1331.05)	475.28 (236.20, 696.67)	0.0001	534.9 (295.7, 780.4)	693.55 (283.33, 1144.21)	0.776

### Propensity Score Matching to Control for Confounding Factors

Propensity score matching was used to “correct” the estimation of the effect of the program on the cardiovascular biomarkers by controlling for the existence of confounding factors in the successful and unsuccessful group. The average treatment effects based on propensity score matching are shown in Table 7. Compared to unsuccessful patients, successful patients who completed the 8-week program had a significant average reduction in PAI-1 [ln transformed concentration: 2.03 ng/mL (95% CI: 1.02–2.7 ng/mL); absolute concentration: 9.07 ng/mL (95% CI: 2.78–15.35 ng/mL); *p* = 0.005], PDGF-beta-1 [ln transformed values: 5.93 pg/mL (95% CI: 3.80–6.56 pg/mL), absolute concentrations (376.077 pg/mL (95% CI: 44.69–707.46 pg/mL); *p* = 0.026); Ang-1 [ln transformed values 7.01 pg/mL (95% CI: 1.07–7.69 pg/mL), absolute concentrations (1104.11 pg/mL (95% CI: 2.92–2205.30 pg/mL); *p* = 0.049)].

**Table 7:** Average treatment effect (defined as difference between first time point and second timepoint) based on propensity score matching (*n* = 52).

	**Coefficient**	**Std error**	***z***	***P*-value**	**95% Confidence interval**
PAI-1 delta	2.03	1.16	2.83	0.005	1.02–2.7
PDGF-beta-1 delta	5.93	5.13	2.22	0.026	3.80–6.56
ANG-1 delta	7.01	6.33	1.97	0.049	1.07–7.69

*Values expressed in natural log scale*.

## Discussion

In this study, we performed a comprehensive analysis of the effects of a short-term exercise programs on traditional and non-traditional risk factors including 184 biomarkers associated with cardiovascular risk. We found that patients engaged in a supervised, remote-monitoring program compared to those participating in supervised in-person traditional programs or unsupervised programs were the most successful in achieving modest improvements in weight loss (≥5 pounds), physical fitness (≥0.5 METs), and/or physical activity (≥1,000 kcals/week). Successful patients not only improved their blood pressure and cholesterol profile, but also lowered the following non-traditional biomarkers of cardiovascular risk: PAI-1, PDGF-beta-1, and ANG-1. Interestingly, C-reactive protein and other non-traditional risk factors including other factors involved in regulating inflammation, coagulation, and metabolism showed no significant change after exercise.

The safety and effectiveness of remote monitoring programs have been demonstrated previously in several observational and randomized trials (3). In at least 20 studies directly comparing home-based programs with traditional in-person programs, remote-monitoring programs were shown to be non-inferior to traditional-in-person programs in reductions in morbidity and mortality and improvement in weight and physical activity. In our study, a significantly higher number of patients enrolled in the home-based program were successful than those in the traditional programs, which is likely related to program adherence.

In addition to improvement in cardiac risk factors in patients enrolled in short-term exercise programs, we demonstrate that modest gains in weight, physical fitness, and/or activity can reduce PAI, PDGF-beta-1, and ANG-1.

PAI-1 plays an important role in the fibrinolytic cascade. As an inhibitor of plasminogen activators, it regulates the formation of plasmin and the lysis of fibrin clots. Higher levels of blood PAI-1 have been associated with a higher risk of incident coronary heart disease even after adjustment for cardiac risk factors (OR = 1.46; 95% CI: 1.13, 1.88) (24). Mendelian randomization models also suggest a causal effect of an increased PAI-1 level on coronary heart disease risk in patients without prior heart disease (odds ratio = 1.22 per unit increase of log transformed PAI-1; 95% CI: 1.01, 1.47). Importantly, a recent meta-analysis that included 38 studies and 11, 577 patients with and without prior ASCVD confirmed the association of higher levels of PAI-1 [e.g., a mean difference of 6.11 ng/mL (95% CI, 3.27–8.96, *P* < 0.001) between cases and controls] with the development of major adverse cardiac events including death, myocardial infarction and cerebral vascular events (25). In our study, patients who successfully completed their 8-week exercise program had a >40% decrease in PAI-1 despite modest improvement in their weight, physical activity and physical fitness. While previous studies have also shown a reduction in PAI-1 after 6 months of exercise (26, 27), exercise programs as short as 10 days have shown no benefit (28). Taken together, these findings suggest that the duration of an exercise program seems to be an important factor in mediating reduction in PAI-1 levels and that even modest improvements in weight loss, physical activity and physical fitness through a healthier lifestyle can reduce PAI-1 levels.

In contrast, PDGF-beta-1, which can be secreted from platelets, macrophages, endothelial cells and vascular smooth muscle cells (29, 30), is a primary growth regulatory molecule that modulates vascular smooth muscle migration and proliferation. PDGF-beta-1 has been shown to promote the development of advanced atherosclerotic lesions *in vitro* and in animal models. Administration of PDGF-beta-1 along with interleukin-1B to human aortic vascular smooth muscle cells *in vitro* results in a phenotypic transition from a contractile to a synthetic state, which is a hallmark of atherosclerosis (31). In animal models, PDGF-beta-1 from vascular smooth muscles leads to the accumulation of leukocytes in the adventitia and media, promoting advanced plaque formation (32). Although serum levels of PDGF-beta-1 are increased after strenuous exercise (33), little information is available on the effects of low to moderate intensity exercise on levels of PDGF-beta-1. We observed that patients who lost weight, improved their physical fitness and/or increased their physical fitness had a >20% decrease in PDGF-beta-1 levels. The reduction of PDGF-beta-1 levels is, thus, another potential mechanism by which exercise may mediate cardiovascular benefits.

ANG-1, on the other hand, is an important regulator of angiogenesis and inflammation (34–36). Although its role in vascular remodeling and development is better defined, studies have also shown that ANG-1 directly activates endothelial cells, neutrophils, and monocytes/macrophages toward a pro-inflammatory state (37). Moreover, animal studies have shown that ANG-1 increases the number of innate immune cells as well as their retention in atherosclerotic lesions (38). Finally, ANG-1 may increase the size of atherosclerotic plaques by inhibiting cholesterol efflux from macrophages as well as stimulating their production of pro inflammatory cytokines (39). We found that patients who successfully completed the program had a 39.8% decrease in ANG-1. To our knowledge, this is the first study to measure ANG-1 levels after exercise in humans and demonstrate a reduction with regimented physical activity, providing an additional mechanism in support for regular exercise and its impact on vascular health.

Although it is unknown the exact mechanisms by which exercise mediates reductions in these non-traditional biomarkers, studies have shown that exercise-induced shear stress in the arteries of both contracting and non-contracting tissue alters the function of endothelial cells, which are the major producers of PAI-1, PDGF-beta-1, and ANG-1 (40–42). Exercise may improve endothelial function through the upregulation of athero-protective genes with concomitant downregulation of atherogenic genes (41, 42), such as PAI-1, PDGF-beta-1, and ANG-1. In a previous randomized control trial, for example, exercise was shown to lower PAI-1, but the greatest reduction was found in patients who had 4G/5G polymorphism of the PAI-1 gene, which resulted in higher baseline levels of PAI (43). Further studies are needed to understand how exercise affects the levels of these non-traditional biomarkers.

Unlike other studies evaluating changes in biomarkers after regular exercise, we did not find significant differences in other markers of inflammation including C-reactive protein. In a recent meta-analysis of 3,769 patients from 83 randomized and non-randomized trials that evaluated changes in CRP after at least 2 weeks of exercise training, exercise training was associated with a reduction in CRP even in the absence of weight loss (44). The greatest improvement in CRP, however, occurred in patients who achieved a significant reduction in BMI or percent of fat. The authors further cite several examples where exercise training did not result in a significant change in CRP including a study of 162 individuals engaged in exercise where only those achieving weight loss >6.4 pounds had a notable improvement in CRP and a study in 421 women where change in weight >5.7 pounds but not improvement in physical fitness was associated with a substantial decrease in CRP (45, 46). Similarly, we did not see a significant improvement in CRP in our cohort, given that the median weight loss in our study was −4.6 pounds (IQR: +2 pounds, −11.8 pounds). Alternatively, changes CRP may not be accurately detected because it is an extremely non-specific marker of inflammation. Because the presence of inflammation anywhere in the body can increase CRP levels, significant reductions in CRP induced by exercise and weight loss may be counter balanced by other factors that increase systemic CRP including infection, stress (47), and poor sleep (48). Taken together, these results suggest CRP may be less sensitive in detecting modest improvements in cardiovascular health after exercise than PAI-1, PDGF-beta 1, or ANG-1.

## Limitations

One of the limitations of this study was that patients were able to select their own exercise program and were not randomized. Consecutive recruitment, however, mimics a real world clinical setting where patients choose their own behavioral modification program. Importantly, there were no significant differences in baseline demographic and clinical factors in patients who were successful or unsuccessful in achieving the defined improvement in weight, physical fitness, and physical activity.

A second limitation of the study is the sample size. Although we were able to find significant improvements in PAI-1, PDGF-beta-1, and ANG-1, we may not have been powered to detect differences in other measured proteomic markers. In addition, the majority of patients recruited were male because the VA is the primary recruitment site and men tend to have coronary artery disease at a younger age (< 70), an age bracket that is less frail and more likely to engage in structured exercise programs. Hence, these findings may not be generalizable to the women.

Thirdly, we do not have long-term follow-up on the patients. During the median follow-up period of 1.9 years (IQR: 1.5, 2.3 years), however, no patients died of cardiovascular related deaths. Of the three patients who suffered a major adverse cardiovascular event (e.g., one patient who had a heart attack with subsequent revascularization and two patients who had elective percutaneous coronary interventions), all three had no significant decrease or an increase in PAI-1, PDGF-1-beta, and ANG-1. These data, however, are limited and further investigation is needed.

Finally, changes in these non-traditional biomarkers may be not only due to improvement in weight loss, physical fitness, and physical activity, but also due to changes in traditional risk factors. This finding is not surprising given these factors have also been associated with hypertension (49), dyslipidemia (50), and diabetes (51, 52). Despite these study limitations, we believe our study provides important information on how an exercise program administered in a real-world setting can improve non-traditional markers of cardiovascular risk.

## Conclusions

Patients achieving modest improvement in weight loss, fitness and/or physical activity after engaging in a short-term exercise program had decreases in PAI-1, PDGF-beta-1, and ANG-1, which are associated with impaired fibrinolysis and inflammation.

## Data Availability Statement

The raw data supporting the conclusions of this article will be made available by the authors, without undue reservation.

## Ethics Statement

The studies involving human participants were reviewed and approved by VA Palo Alto and Stanford. The patients/participants provided their written informed consent to participate in this study.

## Author Contributions

GL: data collection and manuscript preparation. XH, OA, and NH: data analysis and manuscript preparation. JH, SM, HF, KC, DL, and VF: data collection. JM: data collection, data analysis, and manuscript preparation. AL and PN: concept, data collection, data analysis, and manuscript preparation. All authors contributed to the article and approved the submitted version.

## Conflict of Interest

The authors declare that the research was conducted in the absence of any commercial or financial relationships that could be construed as a potential conflict of interest.

## Supplementary Material

Supplementary Figure 1 Two-way scatter plot showing the relationship between ETT METs and METS obtained from cardiopulmonary testing. Spearman correlation coefficient (*r*^2^) = 0.315; *p*-value = 0.0384. Open circles: unsupervised baseline; Closed circles: unsupervised post; Gray plus sign: supervised baseline; Black plus sign: supervised post.

Supplementary Figure 2 Two-way scatter plot showing the relationship between biomarkers measured by the OLINK and ELISA assays. Spearman correlation coefficient (*r*^2^) = *r*^2^ = 0.7925, *p*-value < 0.0001 for plasminogen activator inhibitor-1 (PAI-1); Spearman correlation coefficient (*r*^2^) = 0.717; *p*-value < 0.0001 for platelet derived growth factor beta-one (PDGF-beta-1); Spearman correlation coefficient (*r*^2^) = 0.569; *p*-value = 0.0001 for angiopoetin-1 (ANG-1). White Bar: baseline; Gray Bar: post.

Supplementary Table 1 List of cardiovascular biomarkers measured by OLINK.

## Abbreviations

ANG-1: angiopoetin
ASCVD: atherosclerotic cardiovascular disease
METs: metabolic equivalents
PAI-1: plasminogen activator inhibitor-1
PDGF-beta-1: platelet derived growth factor beta-1.
