# Supplementary figures and images for: Modest Gains After an 8-Week Exercise Program Correlate With Reductions in Non-traditional Markers of Cardiovascular Risk
Source: Front Cardiovasc Med. 2021 Jun 17;8:669110. doi: 10.3389/fcvm.2021.669110 (PMC8245677; doi:10.3389/fcvm.2021.669110)

$r^2=0.315$ , p-value 0.0384

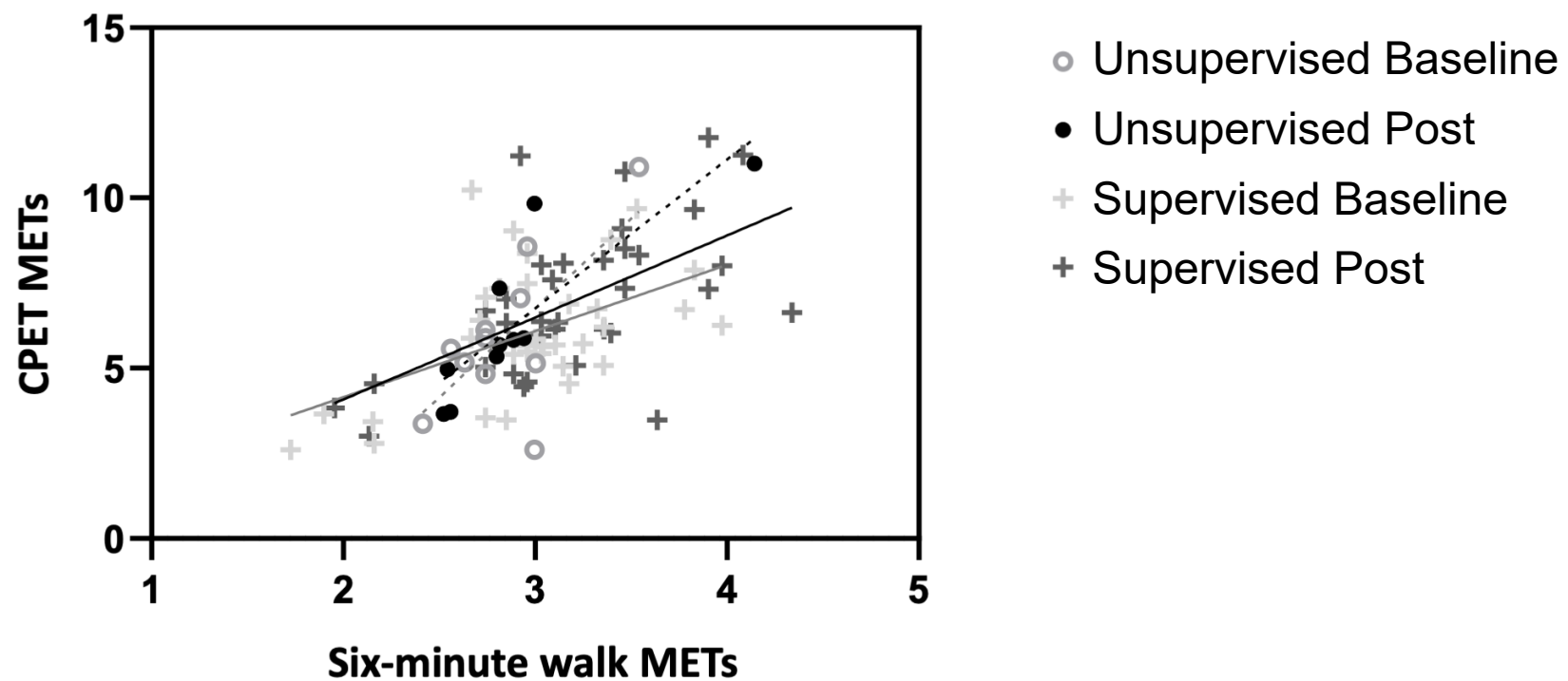

Supplemental Figure 1

Supplement: Supplementary Figure 1 — Two-way scatter plot showing the relationship between ETT METs and METS obtained from cardiopulmonary testing. Spearman correlation coefficient (r2) = 0.315; p-value = 0.0384. Open circles: unsupervised baseline; Closed circles: unsupervised post; Gray plus sign: supervised baseline; Black plus sign: supervised post. [file Image_1.pdf]

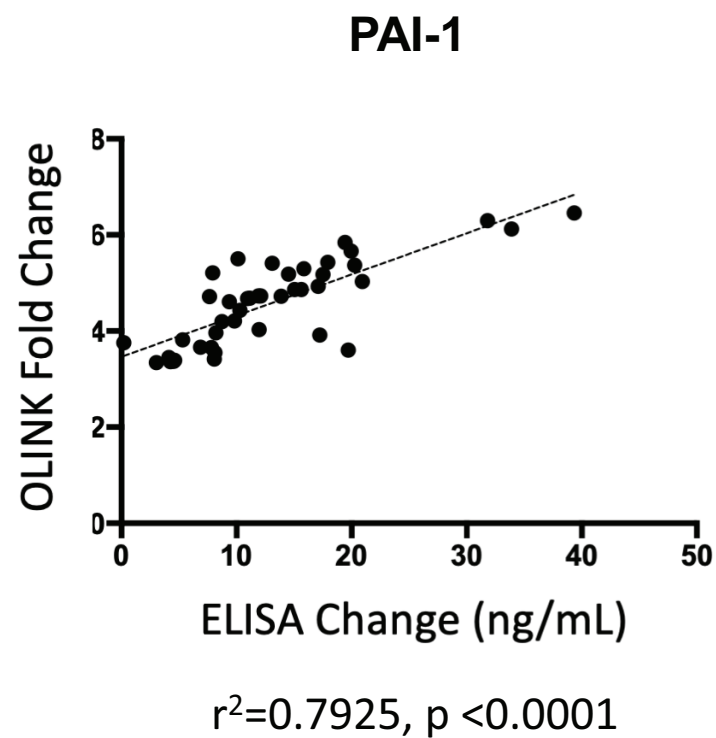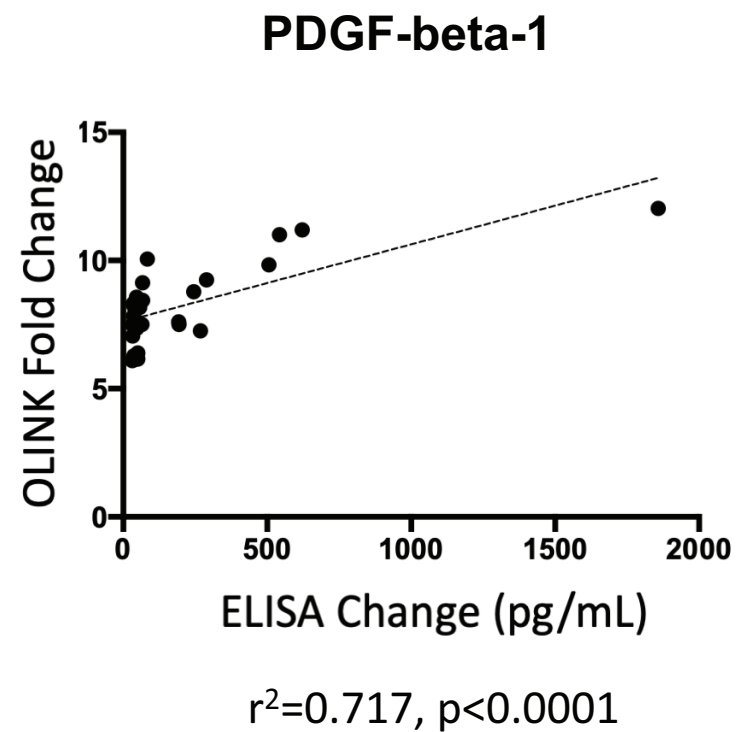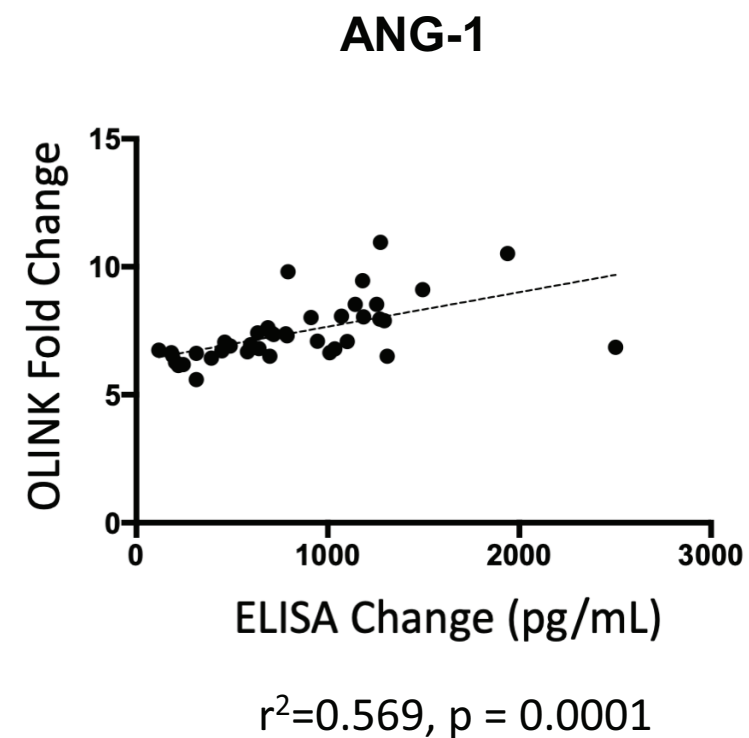

**Supplemental Figure 2**

Supplement: Supplementary Figure 2 — Two-way scatter plot showing the relationship between biomarkers measured by the OLINK and ELISA assays. Spearman correlation coefficient (r2) = r2 = 0.7925, p-value < 0.0001 for plasminogen activator inhibitor-1 (PAI-1); Spearman correlation coefficient (r2) = 0.717; p-value < 0.0001 for platelet derived growth factor beta-one (PDGF-beta-1); Spearman correlation coefficient (r2) = 0.569; p-value = 0.0001 for angiopoetin-1 (ANG-1). White Bar: baseline; Gray Bar: post. [file Image_2.pdf]
